# Advanced sol–gel process for efficient heterogeneous ring-closing metathesis

**DOI:** 10.1038/s41598-021-92043-z

**Published:** 2021-06-15

**Authors:** Shiran Aharon, Dan Meyerstein, Eyal Tzur, Dror Shamir, Yael Albo, Ariela Burg

**Affiliations:** 1 Chemical Sciences Dept, Ariel University, Ariel, Israel; 2 Chemical Engineering Dept, Sami Shamoon College of Engineering, Beer Sheva, Ashdod, Israel; 3 Chemistry Dept, Ben-Gurion University of the Negev, Beer-Sheva, Israel; 4 Nuclear Research Centre Negev, Beer-Sheva, Israel; 5 Chemical Engineering Dept, Ariel University, Ariel, Israel

**Keywords:** Heterogeneous catalysis, Organocatalysis

## Abstract

Olefin metathesis, a powerful synthetic method with numerous practical applications, can be improved by developing heterogeneous catalysts that can be recycled. In this study, a single-stage process for the entrapment of ruthenium-based catalysts was developed by the sol–gel process. System effectiveness was quantified by measuring the conversion of the ring-closing metathesis reaction of the substrate diethyl diallylmalonate and the leakage of the catalysts from the matrix. The results indicate that the nature of the precursor affects pore size and catalyst activity. Moreover, matrices prepared with tetraethoxysilane at an alkaline pH exhibit a better reaction rate than in the homogenous system under certain reaction conditions. To the best of our knowledge, this is the first study to present a one-step process that is simpler and faster than the methods reported in the literature for catalyst entrapment by the sol–gel process under standard conditions.

## Introduction

Olefin metathesis is a fundamental chemical reaction involving the rearrangement of carbon–carbon double bonds that can be used to couple, cleave, ring-close, ring-open, or polymerize olefinic molecules^1–3^. An efficient, powerful, mild, versatile, and selective method, olefin metathesis is used in research in a variety of life sciences, including those with applications in the polymer and pharmaceutical industries^1–27^. Indeed, this method so revolutionized the different fields of synthetic chemistry that the 2005 Nobel Prize in Chemistry was awarded to Yves Chauvin, Robert H. Grubbs, and Richard R. Schrock “for the development of the metathesis method in organic synthesis"^14^.

Olefin metathesis reactions require a catalyst^28^, for example, the ruthenium-based catalysts (a second-generation Grubbs catalyst and a second-generation Hoveyda-Grubbs catalyst) used in this study^3,6,15,18,25–27^_._ Due to catalyst significance, much research has been done to develop an efficient catalyst and efficient catalytic processes. Commonly, homogenous catalysis^4,6,15,18,21–23,25,26^ has been used; however, owing to their high costs^29^, the ability to recycle the catalyst is very important. As such, it is more efficient and productive to use the catalysts as part of a heterogeneous system, which enables them to be recycled. Moreover, the heterogeneous system will not only facilitate easy separation of the catalyst, it will also enable the by-products to be easily recovered from the reaction products. This capacity is of great importance, especially in pharmaceutical production, wherein the final products must meet stringent purity criteria^30–33^.

**Figure 1:**
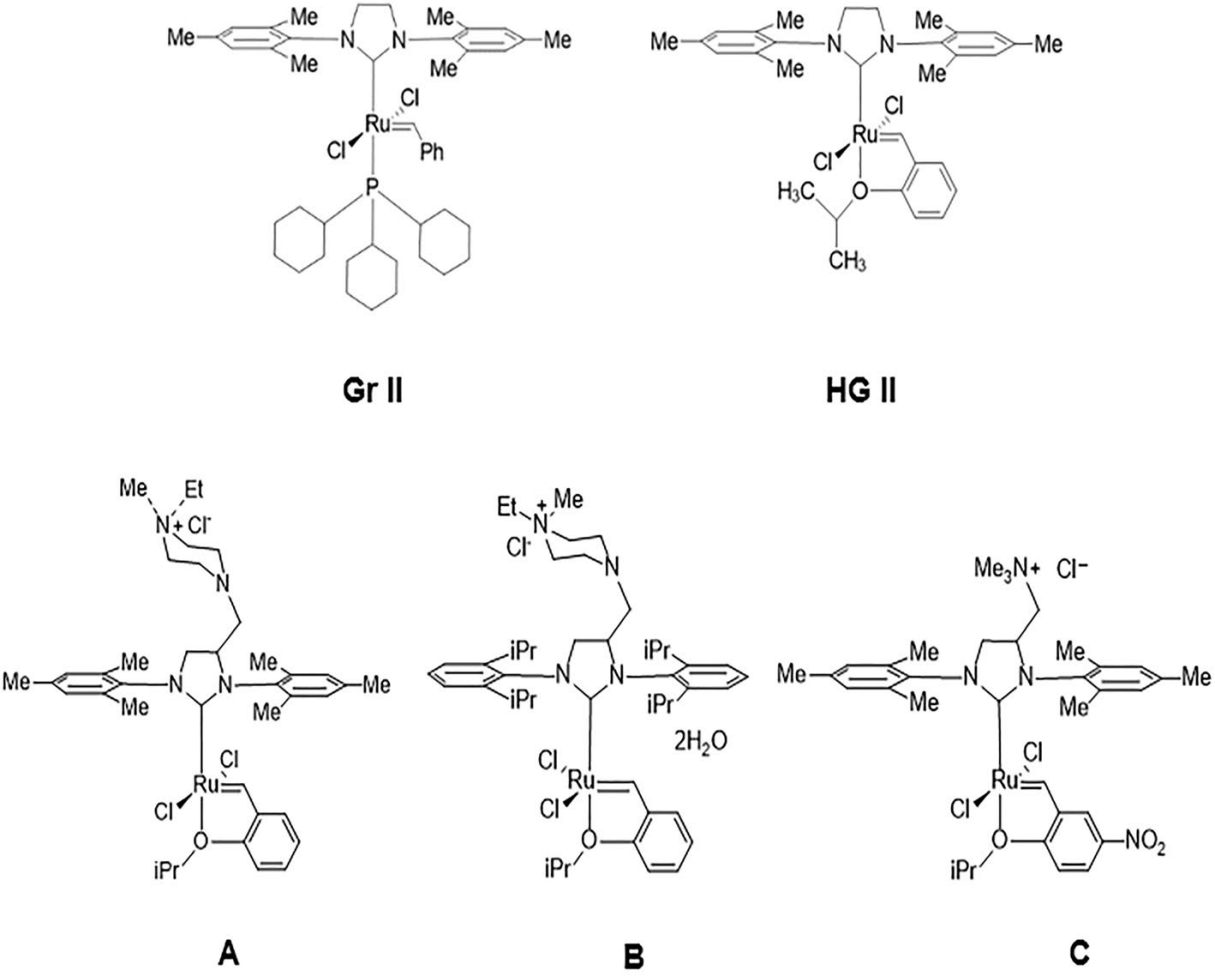
Catalysts used in this study. Type 1—Gr II: Grubbs second generation catalyst; HG II: Hoveyda-Grubbs second generation catalyst. Type 2—(**A**) [1,3-Bis(2,4,6-trimethylphenyl)-4-[(4-ethyl-4-methylpiperazin-1-ium-1-yl)methyl]imidazolidin-2-ylidene]-(2-i-propoxybenzylidene)dichlororuthenium(II) chloride AquaMet; (**B**) (1,3-Bis(2,6-diisopropylphenyl)-4-((4-ethyl-4-methylpiperzain-1-ium-1-yl)methyl)imidazolidin-2- ylidene)(2-isopropoxybenzylidene)ruthenium(II)chloride dihydrate FixCa; and (**C**) 1,3-Bis(2,4,6-trimethylphenyl)-4-[(trimethylammonio)methyl]imidazolidin-2-ylidene]-(2-i-propoxy-5-nitrobenzylidene)dichlororuthenium(II) chloride nitro-StickyCat Cl.

A heterogeneous catalytic system can be created via several routes. One is to fix the ruthenium-based catalysts to a support material, such as mesoporous silica, using the catalyst ion ligand (for example, see catalysts A-C in Fig. 1)^34–37^. Another way is to use the sol–gel process. In the sol–gel process, a porous matrix is formed by mixing precursors such as tetramethyl orthosilicate (TMOS) and tetraethyl orthosilicate (TEOS) and water to produce a 3D inorganic network. A major advantage of the sol–gel process is its ease of adaptability: matrix properties, including particle size and surface area, can be easily and inexpensively controlled by changing the nature and the concentration of the precursors and the pH of the water used in the sol–gel process^30,31,38–52^. The sol–gel process enables the entrapment of a large variety of reagents in the matrices including inorganic molecules^40^, metal nanoparticles, metal-oxide nano-particles^42,44,53–56^, bacteria^57^, and enzymes^45,58^.

The immobilization of ruthenium-based catalysts through a covalent bond, which has been done in several studies^2,47,59^, entails binding the catalyst to the sol–gel matrix. Insofar as the binding process comprises many synthetic stages; however, in this study, we sought to develop a simpler method of catalyst confinement that does not involve covalent binding to the matrix^2,47,59^. Instead, our method relies on intermolecular bonds, which are based on the physical properties of both the catalyst and the matrix. Its successful application has been precluded thus far by the tendency of matrices prepared via this method to exhibit catalyst leakage^30,31,39–44,48,49^, a drawback that we worked to avoid.

In the current study, we entrapped two different types of ruthenium-based catalysts, Fig. 1. Type 1, neutral catalysts, comprised Grubbs second-generation catalysts and Hoveyda-Grubbs second-generation catalysts (i.e., Gr II & HG II). Type 2 were ruthenium-based cationic catalysts, i.e., A-C, in Fig. 1, which are water-soluble due to their aqueous quaternary ammonium group^60–63^.

Catalyst activity and leakage were measured by the conversion of DDM (diethyl diallylmalonate) in a RCM (Ring Closing Metathesis) reaction (reaction 1). The RCM of DDM is often used as a benchmark metathesis catalyst comparisons^64^.
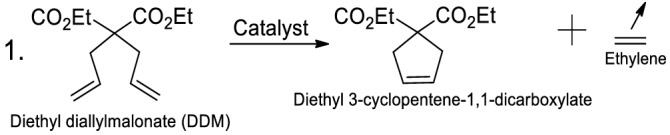


## Experimental section

Type 1 catalysts comprised the Grubbs second-generation catalyst (Gr II); Hoveyda-Grubbs second-generation catalyst (​​HG II); Tetramethyl orthosilicate (TMOS); Tetraethyl orthosilicate (TEOS); Diethyl dialylmalonate (DDM); Methylene Chloride (Dichloromethane, DCM); Toluen; NaOH; HNO_3_ were purchased from Aldrich and were of analytical purity.

Type 2 catalysts comprised the following—**A:** [1,3-Bis (2,4,6-trimethylphenyl)-4-(4-ethyl-4-methylpiperazin-1-ium-1-yl) methyl [imidazolidin-2-ylidene] i-propoxybenzylidene) dichlororuthenium (II) chloride AquaMet; **B:** (1,3-Bis (2,6-diisopropylphenyl) 4-ethyl-4-methylpiperzain-1-ium-1-yl (methyl-imidazolidin-2-ylidene) (2-isopropoxybenzylidene) Ruthenium (II) chloride dihydrate FixCa; and **C:** 1,3-Bis (2,4,6-trimethylphenyl) 4-[trimethylammonio methyl] imidazolidin-2-ylidene] (2-i-propoxy-5-nitrobenzylidene) dichlororuthenium (II) chloride nitro- StickyCat Cl, all of analytical purity, were purchased from Strem Chemicals, Inc.

All water used in this research was ultrapure water, purified by a Treka type TKA-GenPure system with a final resistance of 18.2 MΩ∙cm. All matrices used in this research were prepared according to a procedure published in the literature^30,31,39–44,48,49^. Changes were made to adjust the matrix to our conditions (additional information in SI, Sect. 1.1).

### GC–MS measurements

Conversion and leakage (indirect test) percentages were measured by using gas chromatography combined with mass spectrometry (GC–MS) from Agilent Technologies GC-7820A. The separation of the gases was done in a capillary cell from Maxima using 99.999% pure helium as the carrier gas. The column used was a J & W HP-5 ms Ultra Inert GC Column (30 m, 0.25 mm, 0.25 μm, 7-inch cage) connected to a 5977B mass spectrometer (MS) detector.

Each matrix contained 1.0.10^–6^ mol of catalyst mixed with 1.0 mL of the solvent (dichloromethane/toluene) which contained the substrate (DDM). If 1% of the catalyst had leaked from the matrix, according to the literature^65^ (where conversion has been reported while using < 1 ppm of catalyst), the metathesis reaction would have occurred outside the matrix, and a product peak in the GC–MS chromatogram should appear. Therefore, the leakage measurement was done in two steps: First, leakage was tested using GC–MS, an indirect test. Second, leakage of those samples that showed good results in terms of conversion and leakage, was tested via ICP. This is a direct measurement of the ruthenium in the solvent in the event that it leaks from the matrix (limit of detection equals 600 ppb of ruthenium).

For additional information about conversion and leakage measurements, see supplementary Sect. 1.2.

### ICP-OES (inductively coupled plasma-optical emission spectrometry) instrument

Direct measurement of ruthenium was done by **ICP-OES**, ARCOS model, from Spectro Corp., using Argon plasma at 6000˚C and CCD detector at the wavelength range from 167 to 766 nm.

### BET (Brunauer–Emmett–Teller) measurements

Surface analysis of the tested matrices was measured by a Quantachrome NOVAtouch LX^3^ surface analyzer (N_2_ at 77 K). The measurement was carried out using nitrogen gas (with 99.999% purity from Maxima), with the specific surface area calculated according to the BET curve.

For some of the matrices tested, the surface area appears to have been below the measurement limit, and therefore, large errors are possible.

## Results and discussion

Sol–gel matrices containing types 1 and 2 ruthenium-based catalysts were made according to the procedure described in our recent studies^30,31,39–44,48,49^ and in the Supporting Information (SI) Sect. 1.1. For additional information about conversion and leakage measurements, see supplementary Sect. 1.2. The hydrolysis and condensation reactions in the sol–gel process are known to be acid or base catalyzed. Therefore, all of the matrices in this study were prepared in acidic media (water at pH 2.5) or in alkaline media (water at pH 12)^38,50,52^. The leakage of the type 1 catalysts (Gr II and HG II) was measured, and low conversion rates were observed (Table. S1). To explain these results, the pore radii range measurements by BET of sol–gel matrices that did not contain the catalysts found that the pore radii were equal to 1.5–1.8 nm, Fig. S1. The three-dimensional sizes of the ruthenium complex HG II published in the literature are estimated to be 1.764 nm × 1.370 nm × 1.047 nm^66^. Hence, the catalyst may be smaller than the pore radii of the sol–gel matrix, which would cause them to leak from the matrix during the washing of the latter before the activity test, thereby resulting in low conversion rates.

In view of the poor conversion and leakage findings of the type 1 catalyst, we decided to focus the remainder of the study on the activity of type 2 catalysts entrapped in sol–gel matrices Fig. 1, A-C catalysts. We expected the quaternary ammonium group in the type 2 catalysts to strongly (but not covalently) bind to the silica surface by adsorption, probably via electrostatic bonds with the silanol groups and/or the oxides on the surface of the sol–gel matrix^34,35,37,67^. This bond should decrease catalyst leakage, even during a metathesis reaction conducted in a polar solvent. The conversion was measured as a function of time in the presence of type 2 catalysts in a homogenous system, Fig. S2. Insofar as the solvent may affect substrate penetration to the matrix, matrix activities were studied using two common solvents^68^—toluene and dichloromethane (the latter of which is a more polar solvent)^69^. The relatively polar solvent should not only increase the adsorption of nonpolar substrate to the catalyst, it may also affect substrate flow between the pores and product exit from the matrix due to the intermolecular bonds that formed between the matrix and the substrate^70,71^. Three type 2 catalysts (catalysts A-C) were tested in homogeneous catalysis. High conversions (> 85%, 60 min, Fig. S2) were obtained in dichloromethane (DCM) and toluene, for catalysts A and B due to their good solubility in these solvents^72^. However, the conversions obtained for catalyst C in toluene were relatively lower than those obtained in a relatively polar solvent (DCM) due to the low solubility of catalyst C toluene^62^.

Since catalyst activity can be affected by several parameters, the heterogeneous catalysis was studied as a function of the following: catalyst, solvent, precursor, pH, and the molar ratio between the catalyst and the substrate. Table 1 shows the conversion and surface area as a function of catalyst type and solvent identity.

**Table 1:** Effects of matrix entrapment of type 2 catalysts (A–C) on conversion and surface area values of the matrix^*^ for its first cycle in dichloromethane (I) or toluene (II) as a solvent^**^.

Catalyst	pH 2.5 (dichloromethane)		pH 12 (dichloromethane)	
	% Conversion	Surface area-BET [m^2^/g]	% Conversion	Surface area-BET [m^2^/g]
A	0	0.13	0	234
B	99	1.5	56	181
C	0	8.3	38	261

Catalyst	pH 2.5 (toluene)		pH 12 (toluene)	
	% Conversion	Surface area-BET [m^2^/g]	% Conversion	Surface area-BET [m^2^/g]
A	0	0.67	15	248
B	56	15	97	252
C	0	37	42	355

*Sol–gel matrix was prepared with TMOS at pH 2.5 or at pH 12. ** RCM of DDM reaction 1 Conditions: Reaction time: 24 h; Catalyst: A-C (catalyst_initial_ = 1.0^.^10^–6^ mol); Molar ratio (between the catalyst and the substrate) 5%.

Max leakage equals 1%, which was found by ICP.

When type 2 catalysts were entrapped in sol–gel matrices, the leakage of the catalyst from the matrix was significantly improved, and higher conversion rates were obtained, compared to these of type 1 catalysts in table S1. The leakage improvement could be due to binding of the quaternary ammonium group, in the type 2 catalysts, to the silica surface by adsorption, probably via electrostatic bonds with the silanol groups and/or the oxides on the surface of the sol–gel matrix^34,35,37,67^. Another indication that catalysts A-C bind to the matrix—i.e., become entrapped within it, thus limiting their leakage – can be found in the surface area results. A surface area comparison of a blank matrix with a matrix containing a catalyst (200–460 m^2^/g compare to 5–330 m^2^/g respectively, Fig. S3) indicates that the matrices that contain catalyst have smaller surface areas. As previously reported^34^, this tendency suggests that catalyst binding to ​​the matrix diminishes the latter’s surface area.

The results indicate that the matrices with the highest surface areas were those that contained catalyst C, Table 1. According to a report in the literature^73^ on the activities of catalysts prepared via the sol–gel process, those with larger surface areas contained more active sites that, in turn, fostered the observed increased catalytic activity. In light of this information, matrices containing catalyst C were expected to have higher conversions than matrices with either catalyst A or B. However, the results obtained show that the most efficient heterogeneous system was that with catalyst B, Table 1. Catalysts A and C exhibited a color change from green to black during the sol–gel process, which itself was black at the end of the matrix preparation. The observed color change in the catalyst indicates that it decomposed during matrix preparation^74^. The black color ultimately assumed by the matrix is indicative of catalyst entrapment and probable catalyst decomposition, which would render it catalytically inactive and explain the poor results we obtained for the matrices containing catalysts A and C, Table 1, these results fit the literature. According to the literature, some conditions cause catalyst degradation^75–77^ ; one of them is alkaline media. Goudreault at el.^75^ have shown that hydroxide ions are a potent disruptor for Ru-catalyzed olefin metathesis. This could explain the low conversion of A and C catalysts that were entrapped in acidic or in alkaline media.

The color of the matrix prepared with catalyst B, in contrast, was green, which indicates that the catalyst was not decomposed during the sol–gel process and is probably due to the exchange of the mesityl group of the NHC (N-heterocyclic carbene) ligand in the bulky DIPP (2,6-diisopropyl-phenyl) group. In addition to its role in stabilizing the catalyst’s active form^35^, mesityl group exchange may also help prevent the decomposition of catalyst B during matrix preparation and could explain the higher activity observed for the matrices with catalyst B compared to those containing catalyst A or C, Table 1.

The study of catalytic activity in two different solvents indicates that catalyst leakage when using toluene as the solvent was negligible compared to that obtained when DCM was used, Table 1. In addition, catalyst solvation was improved in polar solvents. Taken together, these experimental results indicate that the catalyst desorbs from the matrix to the solvent in the more polar DCM. All of the following results are for experiments using toluene as the solvent and catalyst B as the entrapped catalyst.

**Figure 2:**
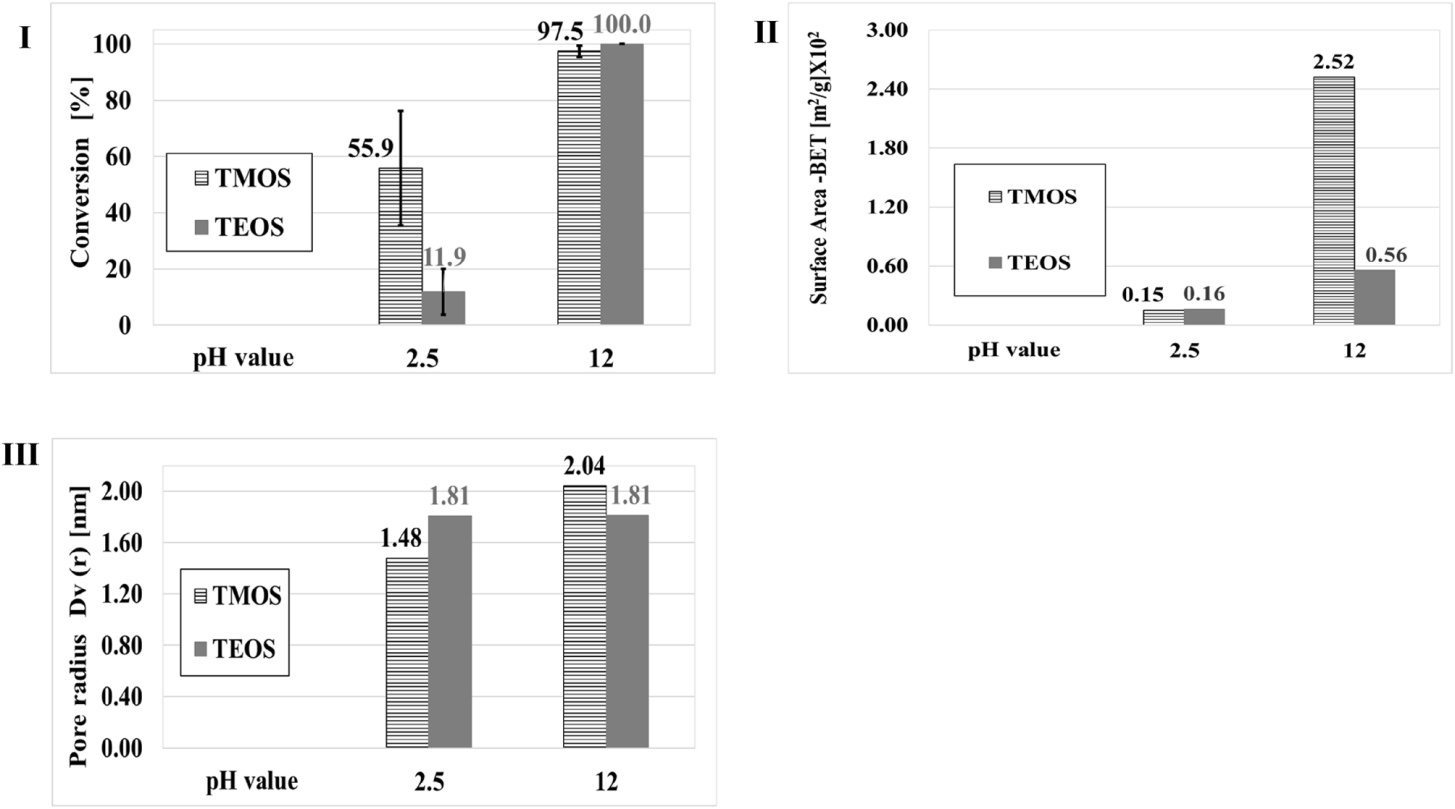
Effects of the pH of the matrices precursors solution on the conversion (I), surface area (II), and pore radii (III) of the matrices for the different precursors in the first cycle, in toluene. *Sol–gel matrix* was prepared with TMOS or TEOS at pH 2.5 or pH 12, Catalyst B (catalyst_initial_ = 1.0^.^10^–6^ mol). *RCM of DDM reaction 1 conditions*: Reaction time 24 h; Molar ratio (between the catalyst and the substrate) 5%. Max leakage equals 1%, which was found by ICP.

The solvent and precursor used to form the matrix are known to affect its skeleton and are therefore expected to affect the catalytic conversion^30,31,38,39,41,48,50,78–80^. Figure 2 shows the conversion, surface area, and pore radii as functions of the pH values for the two precursors TMOS and TEOS.

For the two precursors, preparing the matrix in alkaline conditions yields a more active matrix with a higher surface area than that prepared under acidic conditions, Fig. 2. Hence, the precursor has a smaller influence than the pH on the activity of the prepared matrices. In accordance with reports in the literature^46^, a larger surface area was obtained with TMOS, Fig. 2. The hydrolysis reaction of alkyl silicates under alkaline conditions is influenced mostly by steric effects. Hence, at the same basic pH, the alkyl silicate with the smaller alkyl groups (i.e., TMOS) will react more rapidly with water, resulting in more hydrolyzed species in the water phase. Therefore, it was expected that the skeleton obtained with TMOS would have smaller particles supported by its high surface area^46^. Under acidic conditions, no significant difference was observed between the surface areas of the matrices prepared with TMOS or TEOS. However, the matrix prepared with TEOS had larger pore radii. The existence of larger pore radii when matrix surface areas exhibited no significant difference indicates that the matrix had a smaller number of pores, which implies that it contained less catalyst. Under this scenario, a lower conversion would be expected with the TEOS matrix, which explains the higher conversion obtained for the matrix prepared with TMOS at pH 2.5.

These findings can be explained by the pH of the point of zero charge (PZC) of the matrix. The PZC of silica and silanol precursors is around pH 2–4. At pH < 2, the silica particles have a positive charge, while at pH > 4, they have a negative charge^38,81^. In matrices that are prepared at a pH greater than 4, therefore, it can be assumed that the silica particles will have a negative charge, and as such, they will bind more strongly to catalyst B, a cationic catalyst (containing a quaternary ammonium group). The resulting matrix will entrap/bind more catalyst to its surface than would a matrix prepared at pH < 4. Since higher conversions are obtained when the matrix has been prepared in more alkaline water (pH 12 versus pH 2.5), this expectation is consistent with our results.

**Figure 3:**
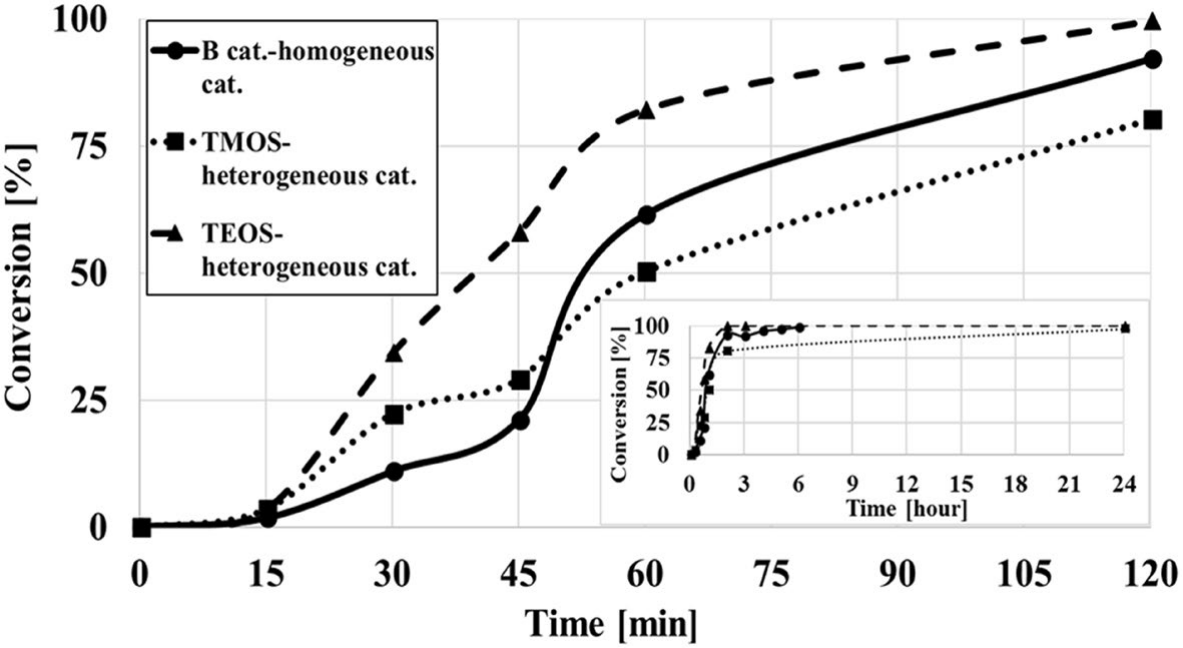
Conversion percentages in toluene as a function of time. *Sol–gel matrix* was prepared with TMOS or TEOS at pH 12, Catalyst B (catalyst_initial_ = 1.0^.^10^–6^ mol). *RCM of DDM reaction 1 conditions:* Molar ratio (between the catalyst and the substrate) 5%; Reaction time: 0–120 min. Figure insert: the same conditions as in Fig. 4, reaction time 0–24 h.

Figure 2 shows that for matrices prepared at pH 12 (with TMOS or TEOS), high conversions (> 95%) were obtained. To optimize the results, the system was studied for its ability to achieve high conversions (above 80%) in shorter reaction times (reaction time < 120 min). The heterogeneous catalytic activity was compared to homogeneous catalysis, Fig. 3.

An induction period can be seen, Fig. 3 and Fig S2, which is common in similar metathesis reactions catalyzed by ruthenium complex^82,83^. This phenomenon occurs in both homogenous and heterogeneous systems. Figure 3 results indicate that the matrix prepared with TEOS had higher catalytic activity than the matrix prepared with TMOS, 80% vs. 50% respectively (after 60 min), B catalyst achieved only 60% conversion in the homogeneous system, after 60 min, Fig. 3. These results indicate that a TEOS-based heterogeneous system is more efficient in an RCM reaction and has shorter reaction times (< 120 min) than the homogeneous system (catalyst B). The results are in agreement with those of previous reports; e.g. Skowerski et al.^35^ has shown that under certain reaction conditions, the heterogeneous catalyst is more efficient than the homogeneous catalyst. Previous studies by Shamir et al.^30^ and Skowerski et al.^35^, have shown that catalyst entrapment in a sol–gel often increases the efficiency of the active species relative to its activity in homogenous catalysis. While it is entrapped in the inner pores of the sol–gel matrix, the catalyst is both protected and stabilized^30^. Frenkel-Mullerad al^58^ illustrated the protective and stabilizing features of sol–gel matrices entrapping the enzyme alkaline-phosphatase, which remained active even at low pH values which in homogeneous catalysis would render it inactive. Another explanation for this tendency could be the specific and rigid geometry of the catalyst in the matrix, which increases the possibility, relative to homogenous catalysis, of it reacting with the substrate.

The control results of pore volumes shown that matrices prepared with TEOS had higher pore volumes than those prepared with TMOS, and exhibit better activity, Figures S4 and 3, respectively. Our results are in agreement with those of previous reports on the binding of these catalysts to other solid supports^34,37^. Pastva et al.^34^ and Kaczanowska et al.^37^ reported that heterogeneous catalyst activity tended to increase with the increase in the pore size of the support used for catalyst entrapment. Our leakage results could also indicate that the quaternary ammonium group of the catalyst is strongly bonded to the matrix by adsorption, probably by contacting the surface silanol groups of the sol–gel matrix.

**Figure 4:**
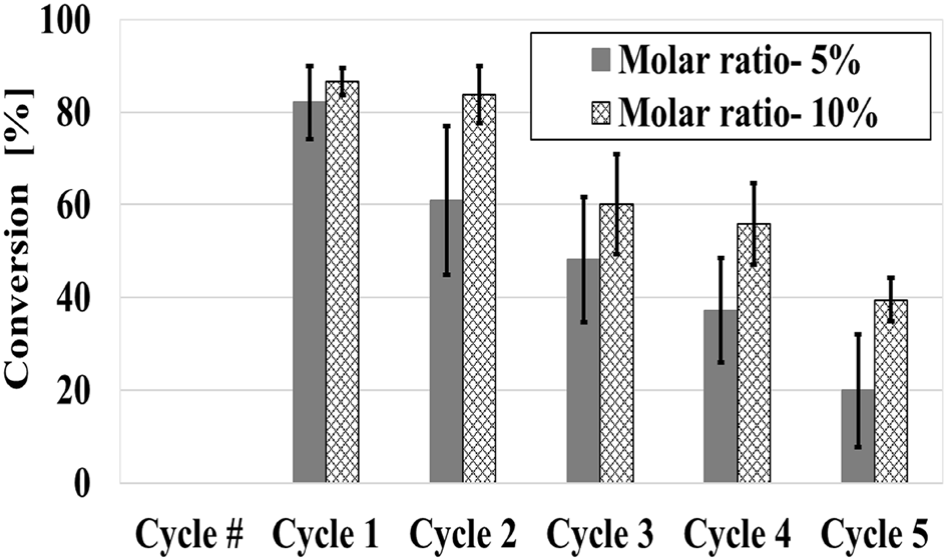
Matrix conversion as a function of the molar ratio and the number of cycles when reaction time is 1 h, in toluene. *Sol–gel matrix* was prepared TEOS at pH 12, Catalyst B. *RCM of DDM reaction 1 conditions:* Molar ratio (between the catalyst and the substrate): at molar ratio 5% catalyst_initial_ = 1.0^.^10^–6^ mol or at molar ratio 10% catalyst_initial_ = 2.0^.^10^–6^ mol; Reaction time: 1 h.

Heterogeneous catalysis enables the reuse of the catalyst, which can reduce the cost of materials, especially when using the expensive ruthenium-based catalysts^29^. Furthermore, the use of a small amount of catalyst multiple times meets the principles of green chemistry^84^. For these reasons, the matrices that exhibited good activity (matrices prepared with catalyst B, at pH 12 and with the precursor TEOS) were tested for their catalytic recyclability when using different molar ratios (i.e., decreased amounts of catalyst) in a reaction time of 1 h, Fig. 4.

The results indicate that the catalyst can sustain good conversion rates for at least five cycles when reused in an RCM reaction. Moreover, larger numbers of cycles are possible when the molar ratio is 10%. After each cycle, the decrease in catalytic activity could be due to catalyst degradation in the reaction work-up^85,86^.

## Conclusion

Studies to develop efficient ruthenium-based olefin metathesis catalysts have been ongoing for over 30 years^3^. Due to the high costs and sensitivity of these catalysts, however, they are typically used in minimal quantities^29,74^. Using them in a heterogeneous system, therefore, which enables the system’s components (e.g., catalyst, by-products, final products, etc.) to be easily separated from each other, could render the process more efficient. But far fewer studies have been done in heterogeneous systems, especially in the simple ones that enable easy separation of the catalyst and the by-products of ruthenium from the reaction products. The ability to separate the components of the system is of great importance, especially in the pharmaceutical industry, where final products must meet stringent purity requirements^30–33^. Therefore, the goal of this study was to develop a simple heterogeneous catalytic process for the entrapment of ruthenium-based catalysts that would be characterized by high efficiency and low catalyst leakage from the matrix. This study focused on finding the optimal conditions in a standard laboratory setting for the entrapment of catalysts in sol–gel matrices without covalent binding, thereby relying only on the physical properties of the catalyst and the matrix. The results we obtained in this study led to the following conclusions:

- Oxide surface area is known to be influenced by the pH^38,40,46,50–52^, and the matrices with the larger surface areas are expected to have more accessible sites, which promote increased catalytic activity^73^. Therefore, sol–gel matrices that are prepared at higher pH values will have higher surface areas and will yield higher conversions. The results indicate that matrices prepared at an alkaline pH are considerably better catalysts than those prepared under acidic conditions.
- The effect on matrix activity of the precursor used was less than the effect of pH. Matrices prepared at the same alkaline pH but with different precursors (TMOS or TEOS) yielded very similar conversions, both of which were higher than those of matrices prepared at an acidic pH. However, changing the precursor material to TEOS yielded matrices with higher pore volumes and better activity in short reaction times.
- Matrices prepared with TEOS at an alkaline pH and using catalyst B as a heterogeneous catalyst yielded better results than the homogenous catalyst B. Moreover, the former is recyclable for at least five cycles in an RCM reaction. The successful optimization of the heterogeneous catalytic process demonstrated in this study laid the groundwork for its future application in the synthesis of important substances in the pharmaceutical industries.

To the best of our knowledge, this study presents a one-step process that is simpler and faster than those reported in the literature^2,47,59^ for catalyst entrapment by the sol–gel process under standard laboratory conditions. This method gives a new and simple means of using a small amount of catalyst and recycling it, thus meeting the principles of green chemistry^84^.

## Supplementary Information

Supplementary Information.
