# Supplementary material for: Advanced sol–gel process for efficient heterogeneous ring-closing metathesis
Source: Sci Rep. 2021 Jun 15;11:12506. doi: 10.1038/s41598-021-92043-z (PMC8206332; doi:10.1038/s41598-021-92043-z)
Supplement: Supplementary file 1 — Supplementary Information. [file 41598_2021_92043_MOESM1_ESM.docx]

**Supporting Information**

**Advanced sol-gel process for efficient heterogeneous ring-closing metathesis**

Shiran Aharon^1, 2^, Dan Meyerstein^1, 3^, Eyal Tzur* ^2^, Dror Shamir^4^, Yael Albo^5^ and Ariela Burg* ^2^

^1^ Chemical Sciences Dept., Ariel University, Ariel, Israel

^2^ Chemical Engineering Dept., Sami Shamoon College of Engineering, Israel

^3^ Chemistry Dept., Ben-Gurion University of the Negev, Beer-Sheva, Israel

^4^ Nuclear Research Centre Negev, Beer-Sheva, Israel

^5^ Chemical Engineering Dept., Ariel University, Ariel, Israel

*Corresponding authors.

E-mail addresses: eyalt@sce.ac.il (E. Tzur), arielab@sce.ac.il (A. Burg).

## *1. Experimental details*

*1.1 Sol-gel matrix preparation*

All of the matrices in this research were prepared according to the procedure published in the literature[1–10]. Changes were made to adjust the matrix to our conditions. Ruthenium-based catalysts (6^.^10^-6^ mol) were weighed with analytical scales and added to an Eppendorf tube. Afterward, quantities of the precursor (140 μL TMOS or TEOS) and the water (70 μL or 45 μL accordingly, at ~~a certain pH value~~ pH 2.5 or pH 12) in r = 4 mole ratio (r = water ⁄ alkoxide) were added to the tube. The contents of the test tube are mixed until one solid phase is obtained. The material is left to age at room temperature until its weight stabilizes, and a sol-gel matrix containing ruthenium-based catalysts is obtained.

Empty sol-gel matrices without the ruthenium-based catalysts are prepared using the same methods.

*1.2 Sol-gel matrix testing*

A sample from the sol-gel matrix was weighed so it will contain 1.0^.^10^-6^ mol of ruthenium-based catalyst. ~~Sol-gel matrix (containing ruthenium-based catalyst) was weighed and~~ The matrix sample was rinsed three times with 1.0 mL of solvent (dichloromethane/toluene).

***Leakage test*** ***(indirect test)***

The matrix sample was rinsed for a fourth time with 1.0 mL of solvent (dichloromethane/toluene), and the separated solvent was transferred to another test bottle to which the substrate (2.1^.^10^-5^ mol diethyl-diallylmalonate, DDM) was added, and the contents were mixed. After several hours (reaction time varies, 0-24 h depends on the experiment) under heating and a nitrogen atmosphere (Solvent: Dichloromethane at 40°C or toluene 80°C), a sample was taken to test leakage by GC-MS. During the leakage test, the substrate did not come into direct contact with the sol-gel but with the liquid that was obtained from rinsing the matrix. Therefore, any catalyst that leaked from the matrix during rinsing would react with the substrate (in RCM of DDM reaction 1), and the product peak would be detected in the GC-MS test. Hence, the catalytic process also involved homogeneous catalysis.

* This test is a quality one: detection of the product peak (reaction 1) in the GC-MS indicates the presence of the catalyst, which has leaked from the matrix, but not its quantitative value.

***Conversion test***

For the conversion test, solvent (1.0 mL of dichloromethane/toluene) and substrate (2.1^.^10^-5^ mol DDM) were added to the rinsed matrix sample, and the contents of the test bottle were mixed. After several hours (reaction time varies, 0-24 h, depending on the experiment) under heating and a nitrogen atmosphere (Solvent: Dichloromethane at 40°C or toluene 80°C), a sample was taken for a conversion test by GC-MS.

***GC-MS method***

Conversion and leakage percentages were measured by GC-MS from Agilent Technologies GC-7820A. The separation of the gases was done in a capillary column using helium gas, 99.999 % from Maxima, as a carrier gas.

The J & W HP-5ms Ultra Inert GC column was used with dimensions 30 m, 0.25 mm, 0.25 μm, 7 inch cage.

The column was connected to a 5977B mass spectrometer detector (MS).

The sample was injected into the device at 80°C, and this temperature was maintained for 2 min, after which column temperature increased every minute for the next 5 min by 30°C until it reached a temperature of 230°C. During this time span, the product's peak (at ~ 5.90 min) and a peak of the substrate (at ~ 6.17 min) were observed. In the next minute, the column temperature rises to 300°C and is maintained for the next three minutes until the end of the run.

***BET (Brunauer–Emmett–Teller ) measurement***

Surface analysis of the tested matrices was done by Quantachrome NOVAtouch LX^3^ surface analyzer (N_2_ at 77 K). The measurement was carried out using nitrogen gas (with 99.999% purity from Maxima), with the specific surface area calculated according to the BET curve.

For some of the matrices tested, the surface area appears to be below the measurement limit, and therefore, there may be large errors.

# ***2. Testing type 1 ruthenium-based catalysts***

*Heterogeneous catalysis of type 1 ruthenium-based catalysts*

The sol-gel matrix containing the type 1 ruthenium-based catalysts (Fig. 1) was added to the reaction vial and mixed with a DDM solution at 40°C, as the solvent was DCM, under nitrogen gas. After 24 h, a sample was taken from the reaction vial for conversion and leakage measurements by GC-MS.

Table S1 shows an example of the activity results for the type 1 ruthenium-based catalysts, the Grubbs second generation catalyst, and Hoveyda-Grubbs second generation catalyst. These results indicate that the prepared matrices had low conversion percentages. Therefore, they could not be reused for several cycles.

**Table S1:** Effect of the pH of the water used to prepare the matrices containing Gr II or HG II catalyst on the conversion in dichloromethane as solvent.

|  | | **Catalyst** | |
| --- | --- | --- | --- |
|  |  | **Grubbs second generation catalyst** | **Hoveyda-Grubbs second generation catalyst** |
|  |  | **% Conversion** | **% Conversion** |
| **pH value** | **2.5** | 0 | 5 |
|  | **12** | 0 | 8 |

***Sol-gel matrix*:** Catalyst: Gr II, HG II (catalyst_initial_ = 1.0^.^10^-6^ mol); Precursor: TMOS; ***Sol-gel matrix*** was prepared with TMOS at pH 2.5 or pH 12.

***RCM of DDM reaction 1 conditions*:** Reaction time: 24 h; Molar ratio (between the catalyst and the substrate) 5 %.

**Note:** leakage not shown in this table as it was less than 1%.

# ***3. Control tests***

*Control test for pore radii dependence on water pH*

To test the effect of the matrix preparation conditions on the pore radii, empty sol-gel matrices (not containing a catalyst) were prepared. Here the pH of the water used to prepare the matrix was tested for its effect on matrix pore radii. Fig. S1 shows the results of this control test for matrices prepared with TMOS or TEOS precursor material.


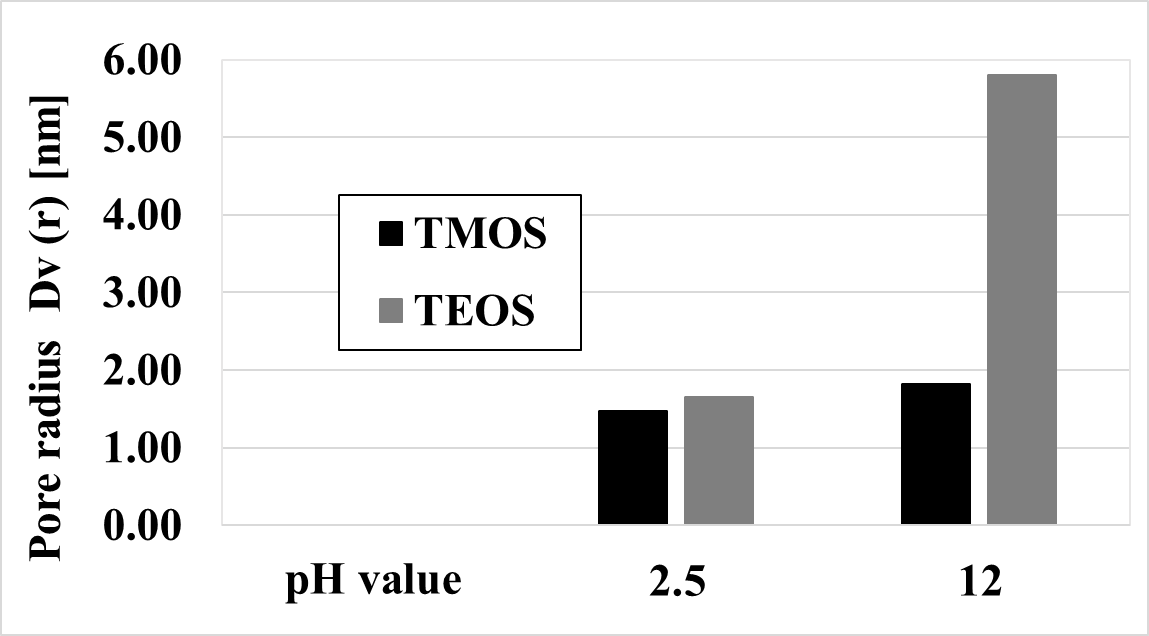


**Figure S1:** Control test for pore radii dependence on water pH for matrices prepared with the precursor TMOS or TEOS. ***Sol-gel matrix*** was prepared with TMOS or TEOS at pH 2.5 or pH 12.

*Control test for homogeneous catalysis*

**Dichloromethane**

**Toluene**


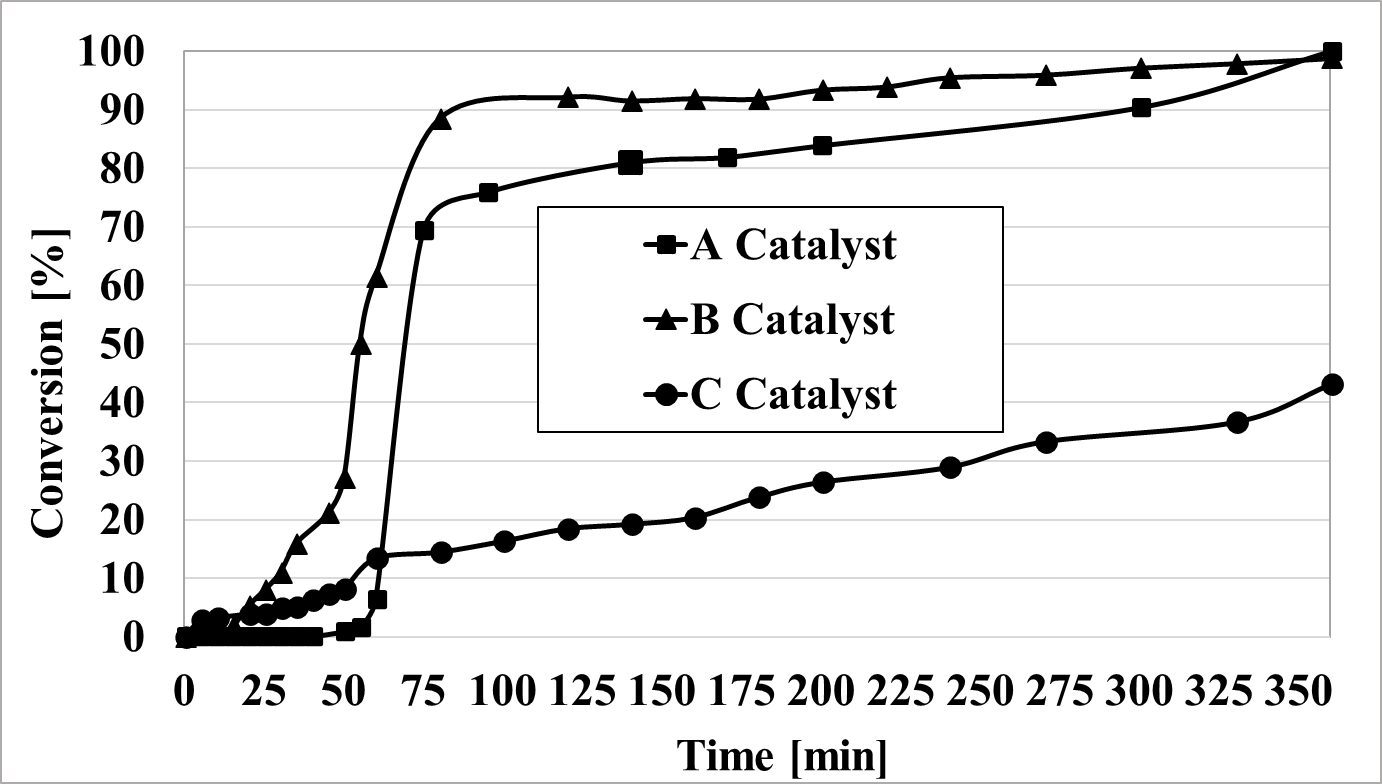

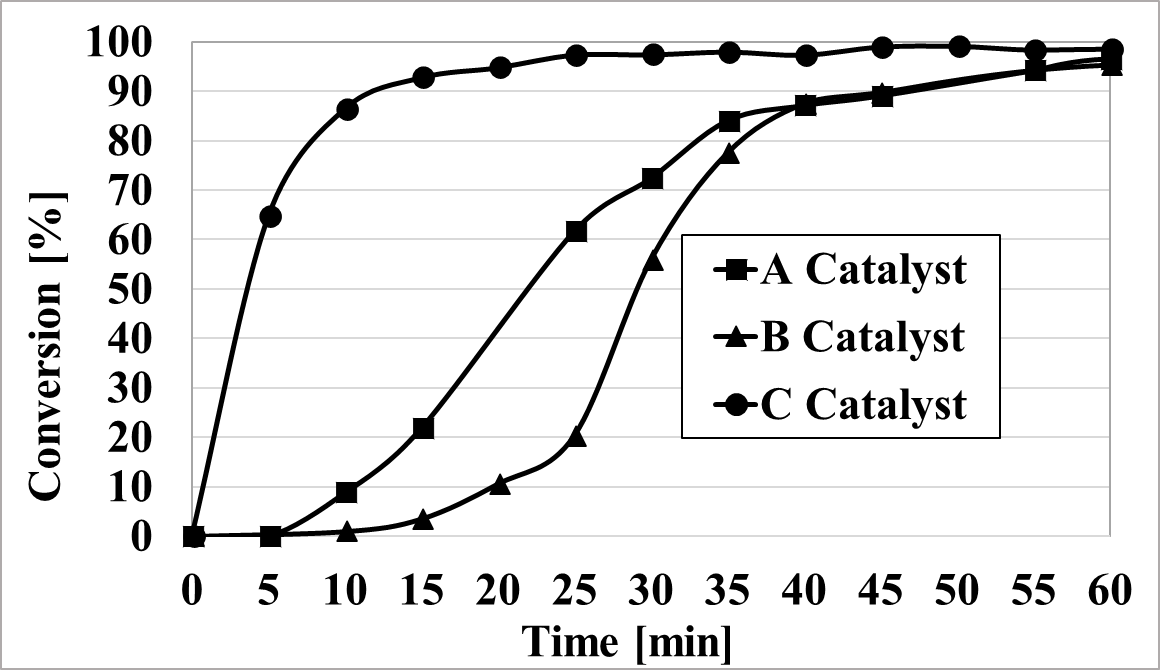


Figure S2: Control test- conversion percentages in dichloromethane /toluene, as time dependent.

***RCM of DDM reaction 1 conditions*:** Catalyst: A, B, or C (catalyst_initial_ = 1.0^.^10^-6^ mol); Molar ratio (between the catalyst and the substrate) 5 %.

*Control test for surface area dependence on the entrapped catalyst*

To test the effect of the entrapped catalyst on the matrix surface area, empty sol-gel matrices (not containing a catalyst) were prepared, and their surface areas were compared to those of matrices prepared with a specific catalyst (catalyst A, B or C).


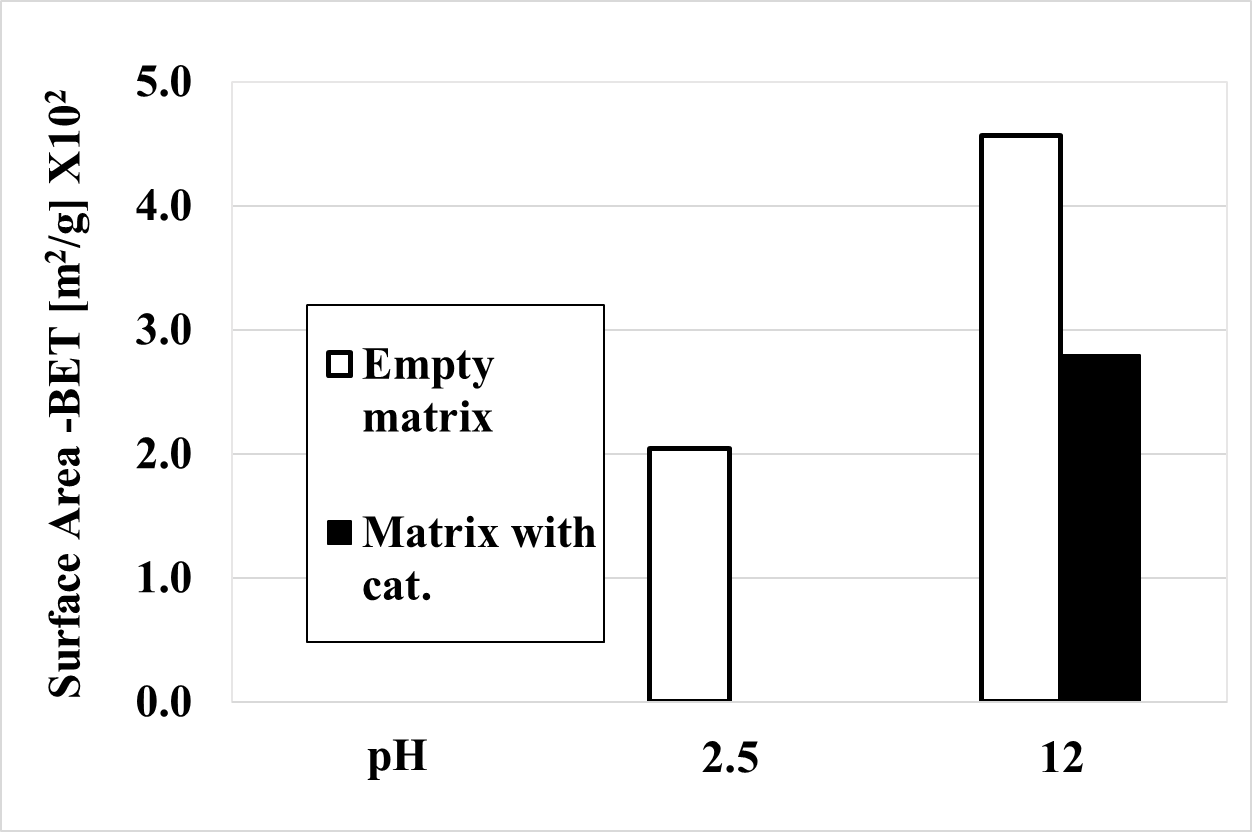


**C cat.**

**B cat.**

**A cat.**


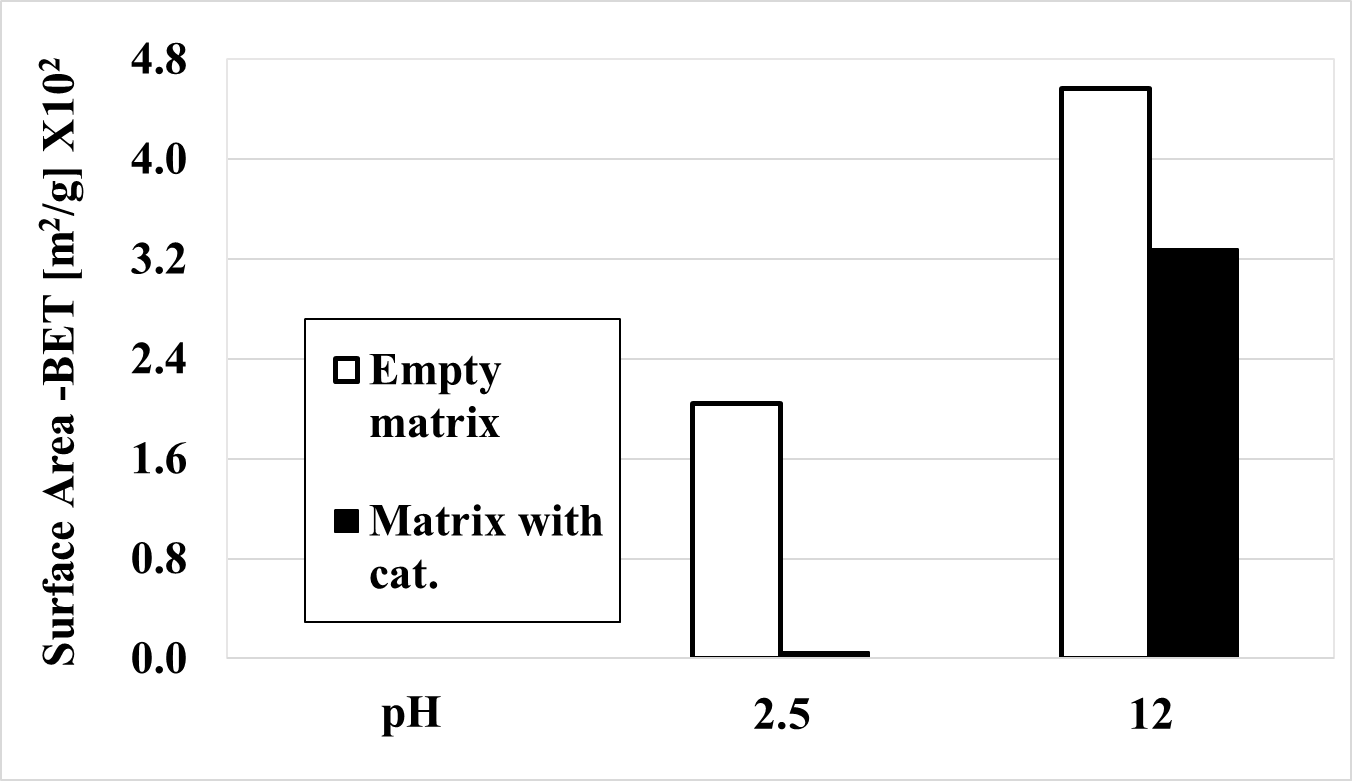

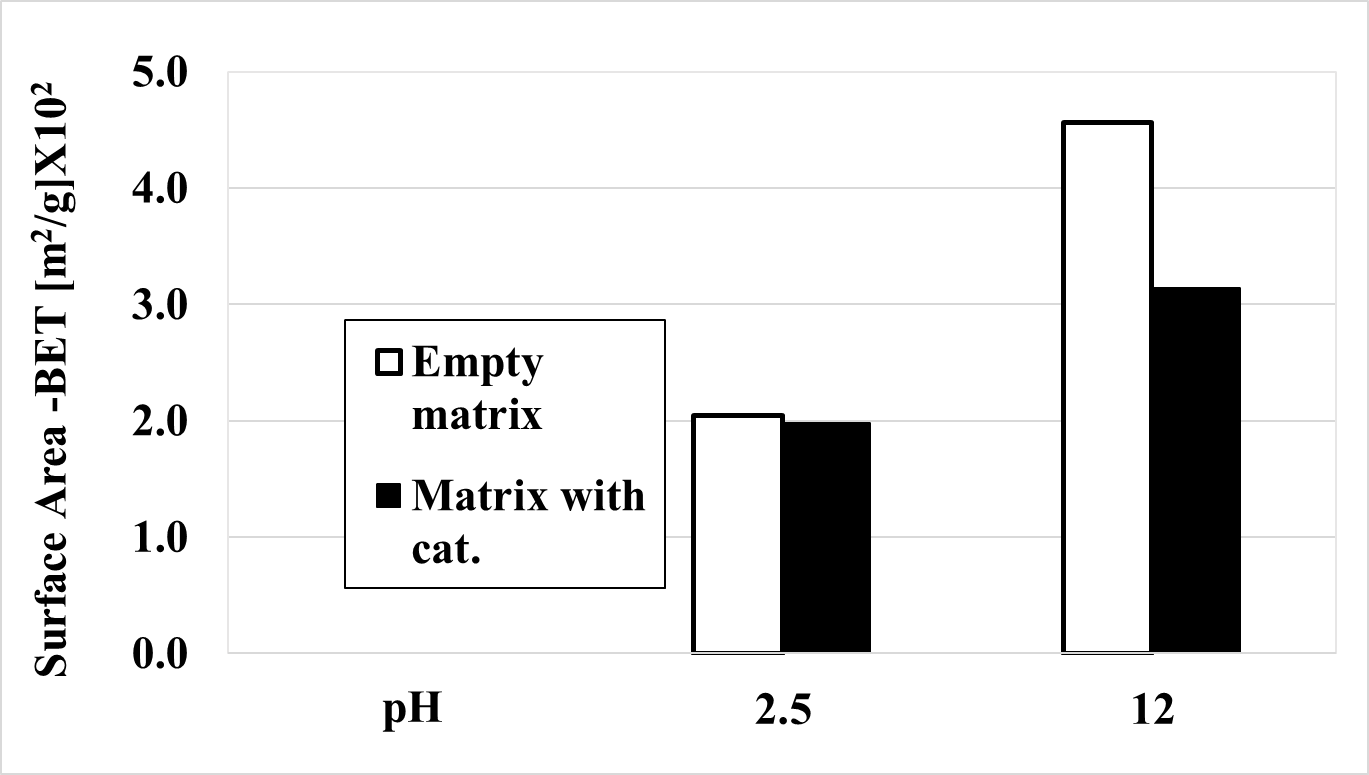


**Figure S3:** Control test for surface area dependence on the entrapped catalyst

***Sol-gel matrix*** was prepared with TMOS at pH 2.5 or pH 12, Catalyst A, B or C.

*Control test for pore volume dependence on water pH*

The effect of the pH of the water used in the preparation of the matrices on the pore volume was measured. For this test, empty matrices were prepared (with TMOS or TEOS as precursor) using water at different pH values. The test results are shown in Fig. S4.

***
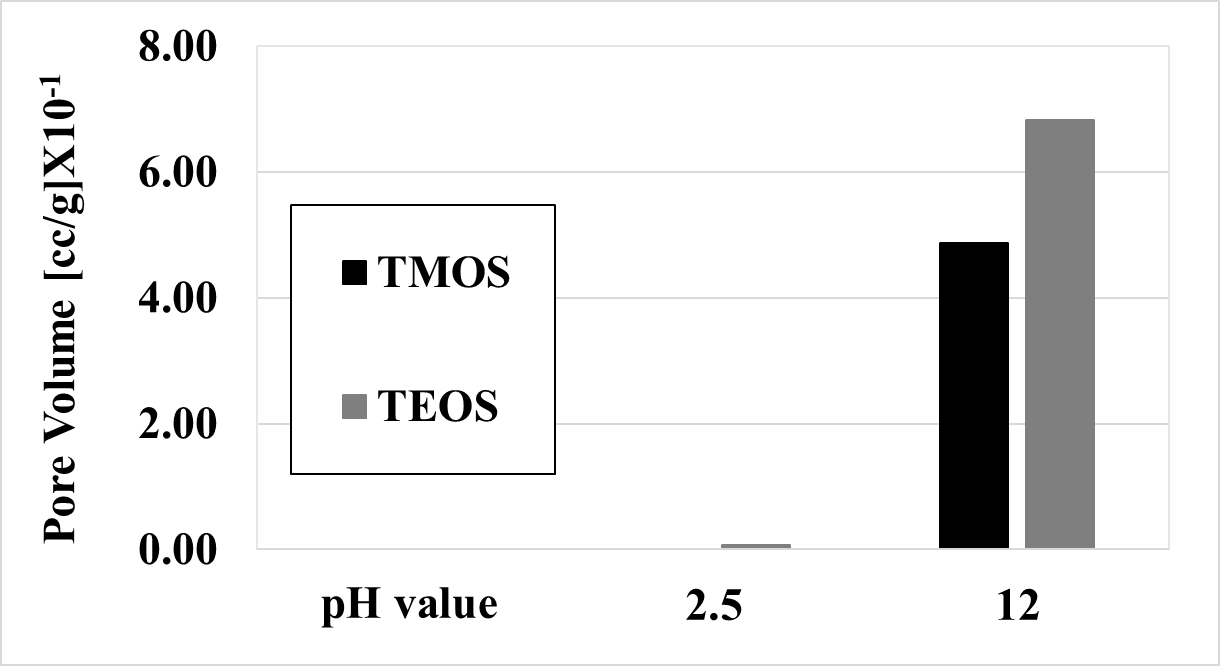
***

**Figure S4:** Control test for pore volume dependence on water pH for matrices prepared with the precursor TMOS or TEOS. ***Sol-gel matrix*** was prepared with TMOS or TEOS at pH 2.5 or pH 12.

**References**

1. Lavi Y, Burg A, Maimon E, Meyerstein D (2011) Electron exchange columns through entrapment of a nickel cyclam in a sol-gel matrix. Chem - A Eur J 17:5188–5192. https://doi.org/10.1002/chem.201003451

2. Neelam, Albo Y, Burg A, et al (2017) Bromate reduction by an electron exchange column. Chem Eng J 330:419–422. https://doi.org/10.1016/j.cej.2017.07.163

3. Neelam, Albo Y, Shamir D, et al (2016) Polyoxometalates entrapped in sol–gel matrices for reducing electron exchange column applications. J Coord Chem 69:3449–3457. https://doi.org/10.1080/00958972.2016.1245418

4. Neelam, Meyerstein D, Adhikary J, et al (2020) Zero-valent iron nanoparticles entrapped in SiO2 sol-gel matrices: A catalyst for the reduction of several pollutants. Catal Commun 133:. https://doi.org/10.1016/j.catcom.2019.105819

5. Neelam, Meyerstein D, Burg A, et al (2018) Polyoxometalates entrapped in sol-gel matrices as electron exchange columns and catalysts for the reductive de-halogenation of halo-organic acids in water. J Coord Chem 71:3180–3193. https://doi.org/10.1080/00958972.2018.1515926

6. Burg A, Shamir D, Apelbaum L, et al (2016) Electrocatalytic Oxidation of Amines by Ni(1,4,8,11-tetraazacyclotetradecane)2+ Entrapped in Sol-Gel Electrodes. Eur J Inorg Chem 2016:459–463. https://doi.org/10.1002/ejic.201500985

7. Shamir D, Wolfer Y, Shames AI, et al (2020) Stabilization of Ni(I)(1,4,8,11-tetraazacyclotetradecane)+ in a Sol-Gel Matrix: It’s Plausible Use in Catalytic Processes. Isr J Chem 60:557–562. https://doi.org/10.1002/ijch.201900139

8. Adhikary J, Meistelman M, Burg A, et al (2017) Reductive Dehalogenation of Monobromo- and Tribromoacetic Acid by Sodium Borohydride Catalyzed by Gold Nanoparticles Entrapped in Sol–Gel Matrices Follows Different Pathways. Eur J Inorg Chem 2017:1510–1515. https://doi.org/10.1002/ejic.201700069

9. Shamir D, Elias I, Albo Y, et al (2020) ORMOSIL-entrapped copper complex as electrocatalyst for the heterogeneous de-chlorination of alkyl halides. Inorganica Chim Acta 500:. https://doi.org/10.1016/j.ica.2019.119225

10. Adhikary J, Meyerstein D, Marks V, et al (2018) Sol-gel entrapped Au0- and Ag0-nanoparticles catalyze reductive de-halogenation of halo-organic compounds by BH4−. Appl Catal B Environ 239:450–462. https://doi.org/10.1016/j.apcatb.2018.08.040
